# Comparison of the Clinical Utility of Two Insulin Resistance Indices: IRI-HOMA and IRI-Belfiore in Diagnosing Insulin Resistance and Metabolic Complications in Children Based on the Results Obtained for the Polish Population

**DOI:** 10.3390/jcm13102865

**Published:** 2024-05-13

**Authors:** Anna Łupińska, Sara Aszkiełowicz, Dorota Kowalik, Krzysztof Jeziorny, Marzena Kolasa-Kicińska, Paula Smalczewska, Arkadiusz Zygmunt, Andrzej Lewiński, Renata Stawerska

**Affiliations:** 1 Department of Endocrinology and Metabolic Diseases, Polish Mother’s Memorial Hospital Research Institute of Lodz, 93-338 Lodz, Poland; anna.lupinska@umed.lodz.pl (A.Ł.); aszkielowiczsara@gmail.com (S.A.); dorota.kowalik96@gmail.com (D.K.); krzysztof.jeziorny@umed.lodz.pl (K.J.); arkadiusz.zygmunt@umed.lodz.pl (A.Z.); andrzej.lewinski@umed.lodz.pl (A.L.); 2 Department of Pediatric Endocrinology, Medical University of Lodz, 90-419 Lodz, Poland; 3 Department of Endocrinology and Metabolic Diseases, Medical University of Lodz, 90-419 Lodz, Poland

**Keywords:** metabolic syndrome, insulin resistance, children, HOMA, Belfiore index, insulin resistance indices, dyslipidemia, obesity, risk factors

## Abstract

**Background:** Recognizing insulin resistance (IR) in children remains challenging due to uncertain IRI-HOMA cut-offs and unclear recommendations for evaluating IR based on OGTT. In our study, we compare the effectiveness of IRI-HOMA and IRI-Belfiore (OGTT-based) in detecting IR and its metabolic complications in children. **Methods:** The analysis included 553 children who were hospitalized at the Department of Endocrinology and Metabolic Diseases of the Polish Mother’s Memorial Hospital Research Institute (PMMH-RI) in Lodz, Poland, between 2002 and 2018 due to various reasons—of these, 67.5% were girls. All underwent OGTT for glucose and insulin assessment. IR diagnosis relied on IRI-HOMA and IRI-Belfiore. IR based on IRI-HOMA was evaluated using three criteria: (A) >2.5; (B) >2.67 in boys and >2.22 in girls before puberty and >5.22 and >3.82 during puberty, respectively; (C) >95th percentile according to charts for IRI-HOMA in children. **Results:** Prepubertal children exhibited significantly lower IRI-HOMA and IRI-Belfiore than their pubertal counterparts (*p* < 0.00005). IRI-HOMA and IRI-Belfiore values positively correlated with age and BMI SDS value (*p* < 0.000001 for all calculations). As many as 26% to 46.9% of children with normal IRI-HOMA showed elevated IRI-Belfiore, with notably higher levels of triglycerides, a lower HDL cholesterol fraction, and a lower HDL/total cholesterol ratio in this subgroup. **Conclusions:** A notable proportion of children exhibited elevated IRI-Belfiore levels despite having normal IRI-HOMA values. This suggests the possibility of peripheral IR preceding hepatic IR in children—omitting an OGTT may therefore lead to overlooking cases of IR. Children diagnosed with IR via OGTT displayed significantly poorer lipid profiles compared to those without IR (characterized by normal values in both IRI-HOMA and IRI-Belfiore). This underscores the ability of OGTT-derived IR indices to identify individuals at risk of developing complications associated with obesity and IR before the onset of metabolic syndrome (MS) symptoms. If IR is already detected in children based on fasting glucose and insulin levels (IRI-HOMA), further evaluation may not be warranted, as OGTT results often simply confirm the diagnosis.

## 1. Introduction

Insulin resistance (IR) denotes the incapacity of normal plasma insulin concentrations to adequately elicit glucose uptake by peripheral tissues, encompassing skeletal muscles and adipose tissue. Furthermore, it leads to an inefficacy in retaining hepatic gluconeogenesis and the release of glucose into the /circulation, as well as suppressing the production of very-low-density lipoprotein [1,2]. IR is most commonly associated with the presence of obesity, type 2 diabetes, dyslipidemia, atherosclerosis, polycystic ovary syndrome (PCOS), and non-alcoholic fatty liver disease [3,4]. Recently, there has been an increasing frequency of IR observed among children and adolescents, which is believed to be strongly correlated with obesity [5]. This is likely linked to reduced physical activity, a sedentary lifestyle, and a high-calorie diet [1]. In obese patients, irrespective of age, there is a significant association between IR and an increased risk of metabolic syndrome (commonly including abdominal obesity, hypertension, and lipid disorders), leading to a higher predicted risk of type 2 diabetes and cardiovascular diseases [6].

However, obesity is not the sole determinant of IR. Among other well-known factors, one should mention the excess of growth hormone (GH) (acromegaly/gigantism) or glucocorticosteroids (Cushing disease/syndrome) or the chronic use of glucocorticosteroids, as well as genetically conditioned IR [1,7,8]. The prevalence of IR varies among different populations, depending largely on ethnicity, genetic factors, and environmental factors, as well as the diversity in methods used to assess IR, cut-off points for numerical values of selected indicators for diagnosing IR, and the methodology employed for data acquisition [1]. In a systematic review study by van Aa et al. [9], the overall prevalence rates of IR ranged between 3.1% and 44% among children and adolescents (both with normal weight and with obesity). Such a large dispersion of results is probably due to the different definitions of IR in the analyzed studies. IR was more frequently observed in girls than boys, likely related to the earlier onset of puberty in girls and the physiologically higher insulin secretion associated with this. It is worth emphasizing that IR can also develop in children with normal body weight; hence, it is essential to identify the best tool for the early detection of IR and its associated consequences.

The gold standard diagnostic method remains the hyperinsulinemic–euglycemic clamp [10]. However, its widespread application is impractical due to technical difficulties and costs. In many studies on obesity or type 2 diabetes, a strong correlation has been observed between the clamp method and mathematical formulas based on the assessment of fasting insulin and glucose levels, such as the Homeostatic Model Assessment of Insulin Resistance (HOMA-IR) [1,5,6].

The medical literature provides various cut-off points for the index of HOMA-IR (IRI-HOMA) for diagnosing IR [9,11,12]. However, the most commonly used cut-off value, adopted in adults and sometimes applied to diagnose IR in the pediatric population, is an IRI-HOMA value > 2.5 [13,14].

In children and adolescents, due to the physiological IR observed during puberty, the interpretation of IRI-HOMA appears to be more complicated. Although different studies have attempted to establish normal values for this model in children and adolescents, reliable reference ranges for IRI-HOMA are not yet available [15,16]. Based on research conducted by Kurtoglu et al. [17], it is assumed that in the pediatric population, IR is indicated by IRI-HOMA > 2.67 in boys and >2.22 in girls before puberty, and >5.22 and >3.82, respectively, during puberty [17]. This suggests that applying cut-offs established for the adult population to children might lead to an overdiagnosis of IR in this age group. Consequently, there is a growing call to interpret IRI-HOMA using percentile charts. Some of these were prepared using data from a large sample—(*n* = 2753) [18] or (*n* = 7074) [19]—of European children [18,19]. One of the authors indicates that high IRI-HOMA is associated with the occurrence of cardiometabolic risk factors, such as increased levels of total cholesterol and triglycerides, low levels of HDL cholesterol, and increased ALT concentration in children [18,19].

Nonetheless, IRI-HOMA primarily gauges hepatic rather than peripheral insulin resistance and, indirectly, insulin sensitivity [20]. Some individuals exhibit heightened and prolonged postprandial insulin secretion, even when their fasting glucose and fasting insulin concentrations are within normal ranges [21]. The assessment of postprandial insulin and glucose concentrations can be effectively carried out during an oral glucose tolerance test (OGTT), such as that proposed by Belfiore at al. [21] (IRI-Belfiore), with modifications tailored for the pediatric population [22]. We chose this formula because it compares the values of the area under the glucose concentration curve and the insulin concentration curve (GLU_AUC_ and INS_AUC_) during the OGTT in the examined patient and compares them with reference values for a given population. IRI-Belfiore shows a relative value ranging from 0 to 2. For the pediatric population, the GLU_AUC_ and INS_AUC_ values of an individual child should be compared to the GLU_AUC_ and INS_AUC_ values of a reference group of healthy children at the same stage of puberty. We developed such reference data in our previous study [22], which allowed us to distinguish physiological from pathological IR. Indicators calculated from the OGTT determine peripheral IR, especially those related to the function of muscles and adipose tissue. However, performing an OGTT with the assessment of glucose and insulin at individual time points is not currently widely recommended for the assessment of IR in either children or adults, although it positively correlates with the hyperinsulinemic–euglycemic clamp method [21]. 

Consequently, the challenge of recognizing IR remains unresolved for two reasons: firstly, due to uncertainties about the cut-off point for IRI-HOMA indicating IR, and secondly, because of the absence of clear recommendations for evaluating IR based on OGTT, which would offer insights into peripheral IR. However, this is an immensely significant issue since identifying IR in a child is linked to the risk of subsequent complications, and diagnosis can serve as a foundation for implementing conservative or pharmacological interventions to prevent their occurrence.

Therefore, the aim of our study was to compare the utility of two IR indices: IRI-HOMA (calculated based on fasting glucose and insulin values) and IRI-Belfiore (calculated based on glucose and insulin concentrations during OGTT) in diagnosing metabolic complications and determining the presence of IR in children.

## 2. Materials and Methods

Approval for the study was obtained from the Bioethical Committee at the Polish Mother’s Memorial Hospital Research Institute (PMMH-RI) in Lodz, Poland.

The analysis included the medical documentation of 553 children aged (mean ± SD): 12.03 ± 4.22 years (min. 2.1 years, max. 17.9 years), involving 374 girls (67.5%) and 180 boys (32.5%), who were hospitalized between 2002 and 2018 at the Department of Endocrinology and Metabolic Diseases of the PMMH-RI in Lodz. Among the examined children, the presence of pituitary adenomas secreting ACTH and/or growth hormone (GH), adrenal adenomas, and the chronic use of glucocorticosteroids were ruled out. During their hospitalization, an OGTT was performed with glucose and insulin assessment at fasting state (0′), and at 60 and 120 min.

**Figure 1:**
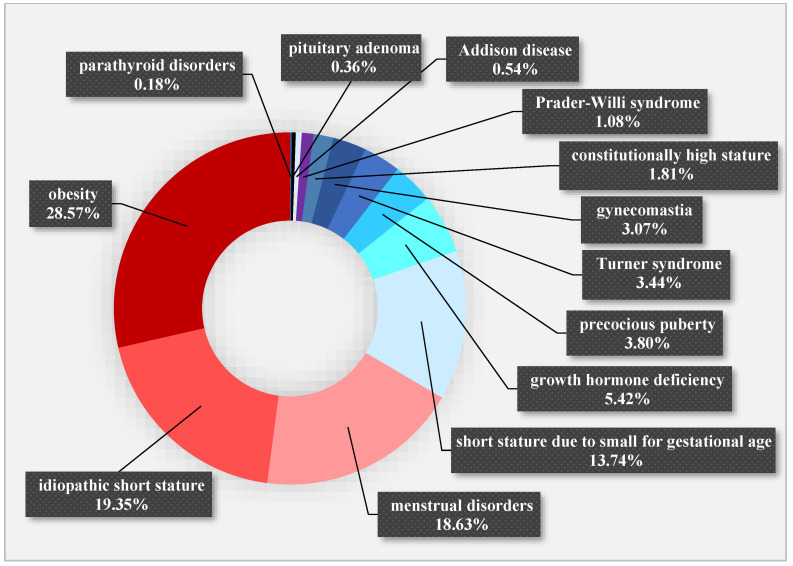
Causes of hospitalization in children examined at the Department of Endocrinology and Metabolic Diseases of the PMMH-RI in the years 2002–2018.

The indication for conducting OGTT was most commonly obesity and an investigation of its complications. Other indications included suspicion of PCOS due to the presence of menstrual disorders and hyperandrogenism, cases of children with short stature preparing for GH treatment, including children with idiopathic short stature (ISS), growth hormone deficiency (GHD), Turner syndrome, Prader–Willi syndrome, or short stature due to being born small for gestational age (SGA), for whom the OGTT is recommended before initiating GH treatment, and children who underwent a GH suppression test (during which insulin levels were also measured) due to suspected or present pituitary adenoma. The complete list of reasons for diagnosing children in the clinic is presented in Figure 1. 

For each child, in addition to the diagnosis, the data of calendar age (CA), height, body weight, and pubertal stage according to the Tanner scale [23] at that time were recorded. Based on this information, the standard deviation score (SDS) for height (hSDS), as well as body mass index (BMI) and BMI SDS values, were calculated using data for the respective percentile charts in the Polish pediatric population [24]. Due to the significant number of children with short stature, the height age (HA) was calculated for each child, and then the BMI SDS values were adjusted for HA (BMI SDS for HA) and not CA in order to avoid distortion of the results. 

OGTT with glucose and insulin assessment was performed for all children. The established standard procedure in our clinic involves conducting this test in a fasting state (after 12 h since the last meal). After obtaining glucose and insulin at time point 0, children are administered glucose at a dose of 1.75 g/kg (up to 75 g) dissolved in 250 mL of boiled water, which the children should drink within 5 min, and glucose and insulin concentrations are measured at 60 min and 120 min after the fluid intake.

For most children, data on TSH, FT4, FT3, ALT, AST, and lipid profile (including triglyceride concentrations, as well as total cholesterol, LDL cholesterol, HDL cholesterol, and calculation of HDL/total cholesterol ratio) during hospitalization were also available.

Obesity was diagnosed if the BMI SDS for HA value exceeded +2.0.

Assessment of IRI-HOMA:

IRI-HOMA was calculated according to the following formula:

IRI-HOMA = (fasting glucose [mmol/L] × fasting insulin [µIU/mL])/22.5) [25]

IR with respect to IRI-HOMA was diagnosed using the three following criteria:

Criterion A: IRI-HOMA > 2.5 [11,13];

Criterion B: IRI-HOMA > 2.67 in boys and >2.22 in girls before puberty, and r > 5.22 in boys and >3.82 in girls during puberty [17];

Criterion C: >95th percentile according to centile charts for IRI-HOMA for children up to 10.9 years old [19].

Assessment of IRI-Belfiore:

Another IR index was calculated based on changes in glycemia and insulin levels during the OGTT, following the method described by Belfiore et al. [21]. IRI-Belfiore was calculated according to the following formula:

IRI-Belfiore = 2/{[1/(INS_AUC_ × GLU_AUC_)] + 1}, where: 

INS_AUC_ = INS_AUCindividual_ /INS_AUCmean_ and GLU_AUC_ = GLU_AUCindividual_/GLU_AUCmean_

GLU_AUCindividual_ and INS _AUCindividual_ represent the area under the curve of glucose and insulin concentration, respectively, during OGTT for a given patient; 

GLU_AUCmean_ and INS_AUCmean_ represent the area under curve of glucose and insulin concentration, respectively, during OGTT for the given population [21]. 

An IRI-Belfiore value exceeding 1.27 was defined as IR [22]. In a prior study, we determined this threshold while evaluating healthy children, achieving a sensitivity of 89.5% and specificity of 89.1% for IRI-Belfiore [22].

All tests were conducted in the same PMMH-RI laboratory in Lodz, using the same method. Plasma glucose was determined by the enzymatic method using hexokinase.

Plasma insulin concentration was measured using the DRG ELISA kit with a sensitivity level of 1.76–100 µIU/mL. The intra-assay coefficient of variation (CV) ranged from 1.8% to 2.6%, and the inter-assay CV ranged from 2.9% to 6.0%.

Descriptive statistics included the number of patients in particular groups and the values of the analyzed parameters, expressed as the mean ± SD. For comparison between different groups, all age- and sex-dependent variables were expressed as SDS values. Student’s *t*-test was applied when the distribution of the variable was normal, while if the distribution was different from normal, a non-parametric statistical test (Mann–Whitney U test or Kruskal–Wallis test) was used for comparisons between groups. Correlations were evaluated using Pearson’s test. Statistically significant differences were accepted when the *p* value was below 0.05.

## 3. Results

In the analyzed group of children, normal body weight was observed in 239 children (43.2%), while obesity was present in 314 of them (56.8%).

Pubertal signs were not observed in 209 children (37.8%) (stage I according to Tanner), early stages of puberty (stages II and III) were assessed in 111 children (20.1%), and an advanced stage or completion of puberty (stages IV and V according to Tanner) was identified in 233 children (42.1%). Pubertal characteristics were therefore present in the majority of children (344 children—62.2%).

There were no differences between the percentage of girls and boys in particular puberty groups or between the normal weight and obese groups. The mean age ± SD of the girls was 12.59 ± 4.21 years; among them, there were 121 (32%) girls without signs of puberty and 253 (68%) with signs of puberty. A total of 154 (41%) girls had normal body weight, while 220 (59%) were obese. The mean age ± SD of the boys was 10.89 ± 4.01 years; among them, 88 (49%) boys were without signs of puberty and 91 (51%) with symptoms of puberty; 85 (47.5%) boys had normal body weight, while 94 (52.5%) were obese.

In the entire analyzed group, IRI-HOMA had a mean ± SD value of 2.6 ± 2.34 SD (ranging from 0.03 to 23.57), while IRI-Belfiore had a mean ± SD value of 1.27 ± 0.39 (ranging from 0.1 to 1.95). 

A comparison of IRI-HOMA and IRI-Belfiore results was conducted based on gender, pubertal stage, and BMI in children. 

### 3.1. Comparison of Two IR Indices Depending on Gender in the Analyzed Group of Children

**Figure 2:**
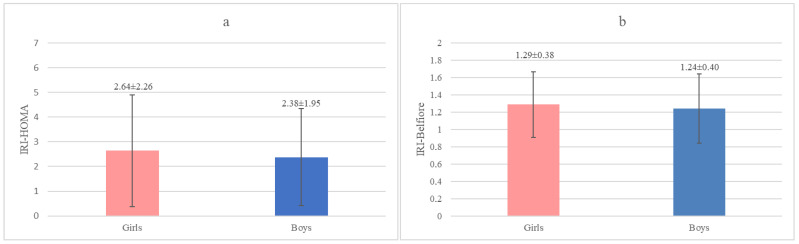
(**a**,**b**) The mean ± SD values of IRI-HOMA and IRI-Belfiore in subgroups of girls and of boys in the analyzed group of children hospitalized at the Department of Endocrinology and Metabolic Diseases of the PMMH-RI between 2002 and 2018 for various reasons.

In the analyzed group of children, neither the mean values of IRI-HOMA nor the IRI-Belfiore values showed significant differences between girls and boys (Figure 2a,b). It seems, therefore, that there is no need to apply separate standards for girls and boys when it comes to IRI-HOMA and IRI-Belfiore.

### 3.2. Comparison of Two IR Indices Depending on Pubertal Stage in the Analyzed Group of Children

**Figure 3:**
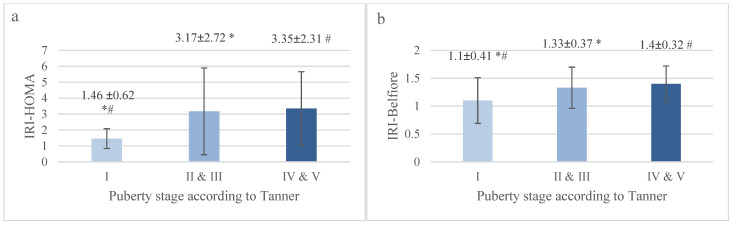
(**a**,**b**) The mean values of IRI-HOMA and IRI-Belfiore in individual subgroups of analyzed children hospitalized at the Department of Endocrinology and Metabolic Diseases of the PMMH-RI between 2002 and 2018 for various reasons, separated according to puberty stage (*#—*p* < 0.00005).

Prepubertal children had significantly lower values of both IRI-HOMA and IRI-Belfiore compared to the group of children in stages II and III of puberty and the group of children in pubertal stages IV and V (Figure 3a,b). Therefore, there is no doubt that the more advanced the puberty stage, the higher the IR indices. For this reason, it seems necessary to develop and apply norms for children at different stages of sexual development.

### 3.3. Comparison of IRI Indices Based on Body Mass Index (BMI) in the Analyzed Group of Children

**Figure 4:**
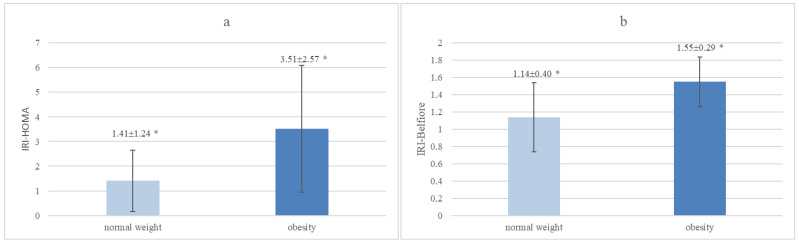
(**a**,**b**) The values of IRI-HOMA and IRI-Belfiore in children with normal weight and with obesity among children hospitalized at the Department of Endocrinology and Metabolic Diseases of the PMMH-RI between 2002 and 2018 for various reasons (*—*p* < 0.000001).

Children with obesity had significantly higher IRI-HOMA values than those with normal body weight (*p* < 0.000001), as well as significantly higher IRI-Belfiore values than children with normal body weight (*p* < 0.000001) (Figure 4a,b). 

Taking into account both body mass index and the stage of puberty, it was observed that in children with normal BMI, IRI-HOMA was significantly lower in prepubertal children than in those in the mid-pubertal stage, and the latter was significantly lower than in children in the advanced pubertal stage. On the other hand, in the case of obese children, IRI-HOMA was significantly lower in prepubertal children than in those in the advanced stages of puberty. However, there was no difference between patients in Tanner stages II and III vs. IV and V of puberty in this regard (Table 1). In each Tanner stage, obese children had significantly higher IRI-HOMA than children with normal body mass.

The IRI-Belfiore values also increased with the advancement of puberty stage and were significantly lower in prepubertal children compared to children in stages II and III and IV and V. This significant difference applied to both the group of children with normal body mass and those with obesity (Table 2). In this case, obese children in different pubertal periods also had significantly higher IRI-Belfiore than children with normal body mass index in the same pubertal stages.

Therefore, regardless of the puberty stage, children with obesity have higher IR indices than children with normal body mass.

### 3.4. Correlations between IR Indices and Calendar Age, as Well as Individual Auxological Parameters and Laboratory Test Results in the Entire Analyzed Group of Children

Statistically significant positive correlations were observed between both IRI-HOMA and IRI-Belfiore and the calendar age (CA) of children and BMI SDS for HA values. Additionally, statistically significant positive correlations were demonstrated between IRI-HOMA and the following biochemical parameters: ALT, AST, triglycerides concentrations, and the concentrations of glucose and insulin at particular time points of OGTT. Negative correlations were observed between IRI-HOMA and HDL cholesterol concentration and the HDL/T–cholesterol ratio. Furthermore, a statistically significant positive correlation was found between IRI-Belfiore and triglycerides, LDL cholesterol, and ALT concentrations, and a negative correlation was found between IRI-Belfiore and HDL cholesterol concentration. A detailed presentation of the observed correlations is provided in Table 3. 

It appears that elevated levels of both IRI-HOMA and IRI-Belfiore correlate with a higher BMI and ALT, and a predisposition towards an unfavorable lipid profile. This inclination towards components of the metabolic syndrome is discernible even in childhood, as is evident from the data analysis of the studied pediatric population.

Although the correlation between IRI-HOMA and IRI-Belfiore is statistically significant, with a coefficient of 0.542, it suggests incomplete alignment between these results in children. Consequently, these indices seem to indicate the presence of a distinct form of IR, implying that the progression of IRI-HOMA might be heralded by the occurrence of peripheral IR identified through IRI-Belfiore.

### 3.5. Frequency of IR Based on IRI-Belfiore

In the entire analyzed group of children, normal values of IRI-Belfiore (defined as <1.27) were observed in 237 children (42.9%), while elevated values of IRI-Belfiore were noted in 316 children (57.1%).

Among the 314 obese children, IR according to IRI-Belfiore was present in 236 of them (75.2% of obese children). It should be emphasized that IR according to IRI-Belfiore was also observed in a significant number of children with normal body weight, as it was present in as many as 80 out of 239 normal weight children (33.5% of them).

### 3.6. Frequency of IR Based on IRI-HOMA According to Different Diagnostic Criteria

**Figure 5:**
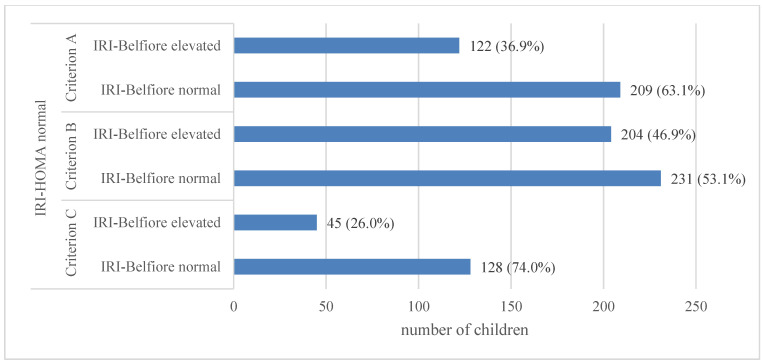

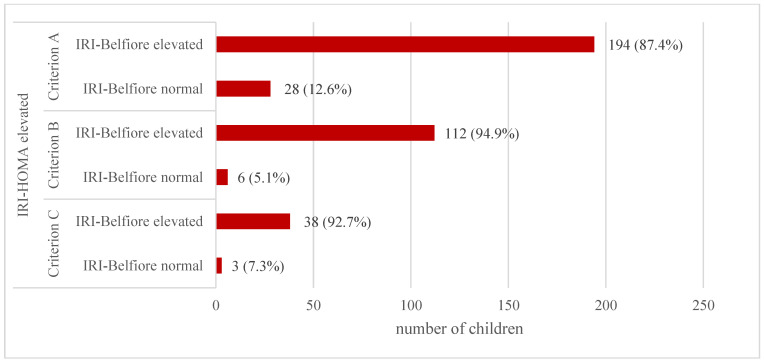
Frequency of IR occurrence based on IRI-HOMA according to criteria A, B, or C and comparison with the frequency of IR diagnosis based on IRI-Belfiore among children hospitalized at the Department of Endocrinology and Metabolic Diseases of the PMMH-RI between 2002 and 2018 for various reasons.

The frequency of IR occurrence based on IRI-HOMA according to criteria A, B or C (see Section 2) and a comparison with the frequency of IR diagnosis based on IRI-Belfiore is shown in Figure 5.

In the entire analyzed group of children, normal values of IRI-HOMA calculated according to criterion A were observed in 331 (59.9%) children, while elevated values were noted in 222 (40.1%) children. Therefore, IR was more frequently diagnosed based on abnormal values of IRI-Belfiore (observed in 316 children, 57.1%) than based on IRI-HOMA according to criterion A. 

IR based on IRI-Belfiore was diagnosed in almost all children with elevated IRI-HOMA and in nearly 37% of those with normal IRI-HOMA (Figure 5). 

The vast majority of children with elevated IRI-HOMA (according to criterion A) had diagnosed obesity (84.7%). On the other hand, every third child with normal IRI-HOMA was obese (38.1%) (Figure 5).

**Figure 6:**
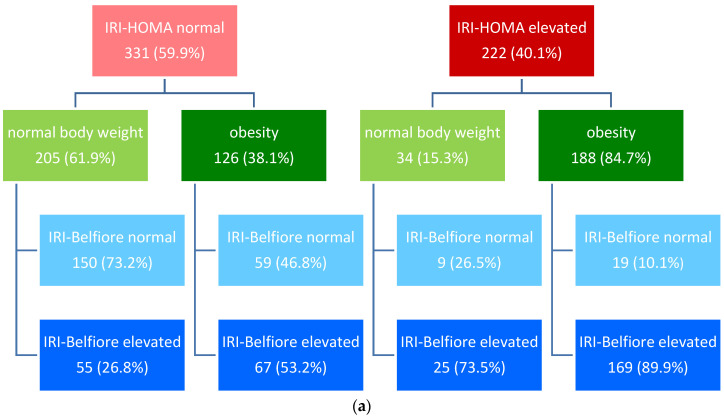

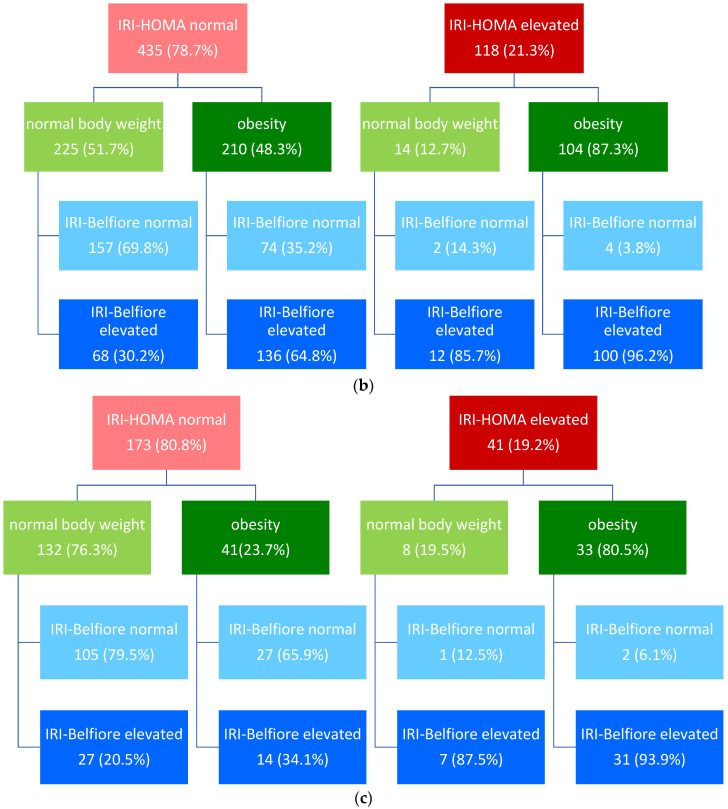
(**a**–**c**) Frequency of IR depending on IRI-HOMA according to criteria A, B, and C and comparison with insulin resistance diagnosed based on IRI-Belfiore among children hospitalized at the Department of Endocrinology and Metabolic Diseases of the PMMH-RI between 2002 and 2018 for various reasons.

In the entire analyzed group of children, a normal value of IRI-HOMA, calculated according to criterion B, was found in 435 (78.7%) children, while an elevated value of the previously mentioned parameter was observed in 118 (21.3%) children (Figure 6b). This means that IR was recognized much less frequently in children according to criterion B than according to criterion A. 

In this case, among children with normal IRI-HOMA according to criterion B, IR was diagnosed based on IRI-Belfiore in almost 47% of cases. If IRI-HOMA was elevated, an abnormal result regarding IRI-Belfiore, confirming the diagnosis, was observed (similarly to the previous analysis) in the majority of cases (94.4%) (Figure 5).

In the analyzed group of children, the assessment of IRI-HOMA according to criterion C was conducted only for children within an age range for which percentile charts are available (*n* = 214 children). Normal IRI-HOMA values calculated according to criterion C were found in 173 (80.8%) children, while elevated values for this parameter were found in 41 (19.2%) children (Figure 6c). This means that IR was diagnosed much less frequently in children according to criterion C compared to criteria A and B. 

It should be emphasized once again that criterion C applies only to children up to 10.9 years of age, i.e., usually before puberty, in whom the incidence of obesity is lower than in adolescents (Table 2), and this may be the reason why IR was diagnosed according to this criterion less frequently than according to criteria A and B.

In this instance, among children with normal IRI-HOMA, according to criterion C, IR was diagnosed based on IRI-Belfiore in only 26% of cases. If IRI-HOMA was abnormal, abnormal results of IRI-Belfiore, confirming the diagnosis, were observed (similarly to the previous analysis) in the majority of cases (92.7%) (Figure 5).

It was observed that, despite having a normal level of IRI-HOMA, nearly half of the examined children exhibited increased insulin secretion during OGTT, resulting in elevated IRI-Belfiore scores (according to both criteria A and B). Among these subgroups, there were statistically significantly higher levels of triglycerides, whereas the HDL/total cholesterol ratio was lower. According to criterion B only the HDL/cholesterol fraction was lower, while the ALT levels were noticeably higher. Additionally, these children had a significantly higher BMI SDS for HA, and they were of advanced age (Table 4).

In children with a normal IRI-HOMA level, performing an OGTT yields valuable supplementary information. Children who exhibit heightened insulin and glucose AUC during OGTT, despite showing normal fasting insulin and glucose results, are often associated with complications linked to excess adipose tissue. These complications may include abnormalities in liver function tests, elevated triglycerides, and HDL cholesterol, predisposing them to develop metabolic syndrome.

On the other hand, in children who had abnormal IRI-HOMA, the majority also had an abnormal result for IRI-Belfiore. These groups did not significantly differ in any of the laboratory parameters indicating metabolic syndrome (Table 5).

Group C was too small to facilitate meaningful comparisons between results; however, it is noteworthy that the few children in the subset with favorable IRI-Belfiore values were significantly leaner than the remaining participants.

## 4. Discussion

IR is one of the major risk factors for the development of type 2 diabetes, arterial hypertension, and dyslipidemia [26]. Research indicates that it serves as a predictor for the development of type 2 diabetes even in patients with normal glucose tolerance. Therefore, it is crucial to recognize IR in the pre-development phase, when medical intervention is most effective [26].

Undoubtedly, IR, in both adults and in the developmental age population, is most commonly associated with obesity and is usually the first sign of impaired glucose metabolism [27]. However, there are reports that IR may also occur in patients with normal body weight [9,28]. We reached the same conclusion based on an analysis of the data from our patients in the current study—according to various indicators of IR and different cut-off points, this affected 12.7% to 19.5% of children according to IRI-HOMA, and 33.5% according to IRI-Belfiore.

In our study, a statistically significant positive correlation was observed between both IRI-HOMA and IRI-Belfiore and the age of children, as well as their BMI SDS values. Peripheral and hepatic IR were therefore more common in older children with excess body weight. A statistically significant positive relationship was also demonstrated between IRIs and the activity of liver enzymes and the concentration of triglycerides, while a negative correlation was found between IRIs and HDL cholesterol, as well as the HDL/total cholesterol ratio. Similar relationships have been reported in the works of other authors [11,27].

In the study by Ballerini et al. [11], it was also found that elevated fasting insulin levels are positively correlated with age, BMI, pubertal stage, IGF-1 concentration, and triglyceride levels [11], similar to the findings in our study. Additionally, it was stated that children aged >7.5 years had higher insulin levels than those <7.5 years. However, the elevated serum insulin concentration in older prepubertal children was not associated with an increase in BMI. In the group of children during puberty, insulin levels were consistently higher than among prepubertal children.

In the study conducted by Lentferink et al. [27], it was observed that children with confirmed IR had a higher BMI SDS. Additionally, patients with IR had statistically significantly higher triglyceride levels and lower HDL cholesterol levels. The authors also demonstrated that gestational age, pubertal development stage, and BMI SDS were positively correlated with IR in younger children (<10 years of age), while in teenagers only BMI SDS was positively correlated with IR. In children under 10 years of age, IR assessed based on IRI-HOMA was more frequently observed in girls. The authors explained this by the fact that puberty in girls begins earlier than in boys. However, in teenagers, no differences in the frequency of IR were observed depending on gender, which was attributed to the increasing degree and frequency of obesity with age in both genders, mitigating the influence of puberty on the development of IR [27]. Goran et al. [29] explained the lack of significant influence of abdominal fat on insulin sensitivity in the younger children by the relatively lower subcutaneous fat content in this age group. 

In our study, we also observed no significant correlation between either IRI-HOMA or IRI-Belfiore and the child’s gender. Nevertheless, both indicators were notably influenced by the child’s age, stage of pubertal development, and BMI. Therefore, obese older children in the pubertal period were more likely to develop both peripheral and hepatic IR. On the other hand, it seems that excess fat tissue may not be the key determinant of IR development in children. Arslanian et al. [30] demonstrated that the frequency of IR in obese teenagers was higher than in obese adults, despite the similar levels of fat tissue content and glycemic status [30]. According to the authors, these results may be associated with the more rapidly progressing disturbances in beta-cell function observed in teenagers compared to adults diagnosed with type 2 diabetes.

The frequency of diagnosing IR is undoubtedly influenced by various criteria beyond the discussed factors determining its development. Another challenge arises from the fact that there is no single biochemical definition of IR in children and adolescents. The most commonly used indicator for recognizing IR is IRI-HOMA, for which the measurement of fasting glucose and insulin is sufficient. In the adult population, IR is often diagnosed if IRI-HOMA > 2.5, but there are proposals to use higher cut-off points for this indicator [9,31]. Some researchers also employ the OGTT as a tool for diagnosing IR. For instance, Ten et al. [32] identify hyperinsulinemia in children when fasting insulin concentration exceeds 15 µU/mL, and the peak insulin concentration is ≥150 µIU/mL and/or insulin concentration > 75 µIU/mL, observed two hours after glucose administration. 

In a study conducted on over 200 healthy children aged 1–18 years, Ballerini et al. [11] highlight that fasting insulin levels > 10 µIU/mL in prepubertal children and 17 µIU/mL for girls and 13 µIU/mL for boys during adolescence may serve as cut-off points for diagnosing IR. Thus, they suggest that IRI-HOMA values > 2.0 for prepubertal children and 2.6 during adolescence are risk factors for the development of IR [11].

The lack of globally accepted criteria for diagnosing hyperinsulinemia and IR in children prompted us to analyze the research results obtained based on three different criteria for diagnosing IR using IRI-HOMA: the classically accepted cut-off for adults (criterion A), the criterion established by Kurtoglu et al. [17] in a large group of studied children (criterion B), and the percentile charts of Peplies et al. [19] (criterion C).

Peplies et al. [19] created percentile grids for prepubertal children based on studies involving over 7000 children with normal body weight aged 3 to 10.9 years from eight European countries. In our study, we also referred to these cut-offs. A IRI-HOMA index > 95th percentile for gender and age served as the basis for diagnosing IR. Applying this criterion (criterion C), we found a normal value of IRI-HOMA in 173 (80.8%) children, while it was elevated in 41 (19.2%) children (in our study, 214 children remained within the age range of up to 10.9 years, allowing the use of these percentile charts). Therefore, IR was detected less frequently in children according to criterion C than according to criteria B and A. This may result from the lower prevalence of obesity in prepubertal children and the absence of the influence of sex hormones on the development of IR. It is worth emphasizing once again that the application of the IR diagnosis criteria adopted for adults may contribute to overdiagnosis in this group of children.

In our study, we observed significant differences in the frequency of IR diagnosis, depending on the indicator used. Regardless of the IR diagnosis criteria based on IRI-HOMA applied in each examined group, approximately 90% of patients with elevated IRI-HOMA simultaneously had IR diagnosed based on IRI-Belfiore (for criterion A: 87.4%; for criterion B: 94.9%; for criterion C: 92.7%). On the other hand, in a considerable fraction of our patients with normal IRI-HOMA, IR could be identified based on IRI-Belfiore (according to criterion A: 36.9%; according to criterion B: 46.9%; according to criterion C: 26.0%). The lowest percentage of IR diagnosis based on IRI-Belfiore in group C most likely stems from the fact that only prepubertal children were included in this group, where, from a population perspective, the prevalence of obesity is lower than in older children (perhaps these are the children in whom obesity has not yet developed, but there are predispositions for its occurrence). At the same time, there was a statistically significant positive correlation between both indicators (IRI-HOMA and IRI-Belfiore).

Lewandowski et al. [12], in their study of a group of adult women with PCOS, also concluded that in the vast majority of patients with elevated IRI-HOMA, IRI-Belfiore was simultaneously elevated—only 4.3–7.25% of patients with elevated IRI-HOMA had a normal IRI-Belfiore value [12]. They observed, however, that a significant number of patients diagnosed with IR based on IRI-Belfiore had a normal IRI-HOMA value, regardless of the cut-off point used to determine this IR index (IRI-HOMA = 3.46 or IRI-HOMA = 3.8) [12]. The authors explain this phenomenon by noting that both indicators assess slightly different types of IR (IRI-HOMA assesses hepatic IR, while IRI-Belfiore determines peripheral IR). This is another indication suggesting that relying solely on IRI-HOMA based on fasting glucose and insulin values does not provide a complete insight into the relationships concerning glucose and insulin secretion. In certain situations, performing a full OGTT with insulin assessment provides new insights into the presence or absence of disturbances in this area.

However, there is a definite relationship between these types of IR. Peripheral IR appears to precede the development of hepatic IR. In a state of chronic caloric excess, body tissues become resistant to insulin signaling. Skeletal muscles are a large reservoir of circulating glucose, responsible for up to 70% of glucose removal, as measured by hyperinsulinemic–euglycemic clamp. The direct consequence of muscle IR is reduced glucose uptake by muscle tissue. Glucose is transported from the muscles to the liver, where de novo lipogenesis occurs. With an increase in glucose substrate in the liver, IR also develops. Higher de novo lipogenesis rates increase serum triglyceride levels, creating an environment with an excess of energy substrate, which increases IR throughout the body, contributing to ectopic lipid deposition in and around the visceral organs [33,34]. 

In our study, a significant proportion of patients with normal fasting insulin levels and normal IRI-HOMA exhibited elevated IRI-Belfiore, confirming that peripheral IR precedes the development of hepatic IR. This group included both patients with normal body weight and patients with obesity. In patients with normal IRI-HOMA (criterion B) and elevated IRI-Belfiore, we observed statistically significantly higher BMI SDS, older age, and higher ALT serum concentration, triglyceride levels, as well as lower HDL cholesterol levels compared to patients without IR (with normal IRI-HOMA and normal IRI-Belfiore). On the other hand, elevated fasting insulin levels were usually associated with higher insulin levels at 120 min of OGTT, as demonstrated in our study. A similar relationship was observed in another study involving prepubertal children who were diagnosed as being born small for gestational age (SGA) [35]. Elevated IRI-HOMA and the development of hepatic IR were largely associated with previously diagnosed peripheral IR (elevated IRI-Belfiore). This issue affected over 80% of obese children with SGA in that study [35].

Therefore, IRI-Belfiore based on OGTT allows for the detection of patients with peripheral IR and increased risk factors for cardiovascular diseases, even before the development of metabolic syndrome and its associated consequences.

Borai et al. [36] indicates that indices based on OGTT have an advantage over these relying solely on fasting glucose and insulin levels in that they can provide and earler indication of a decline in insulin sensitivity, mainly associated with impaired insulin secretion stimulation due to increased peripheral glucose consumption [36]. The authors suggest that OGTT-based indices may be primarily intended as a screening tool for diagnosing IR in children with obesity and a family history of type 2 diabetes and cardiovascular diseases. Al.-Beltagi et al. [1] emphasize that the assessment of peripheral IR can serve as a means of screening to detect IR, especially in patients at high risk of developing type 2 diabetes, despite the absence of clinical risk factors such as obesity and impaired glycemia [1].

One of the risk factors for cardiovascular diseases is disturbances in lipid metabolism, often accompanying obesity [37]. In numerous studies, a relationship between IR and decreased HDL cholesterol levels has been observed [38,39]. This finding aligns with the hypothesis that the production of HDL cholesterol is linked to the degradation of LDL cholesterol particles, which is impaired by IR [40]. In our study, there was also a statistically significant positive correlation between both examined IR indices (IRI-HOMA and IRI-Belfiore) and triglyceride levels, along with an inverse relationship between these IR indices and HDL cholesterol levels. In another study conducted on a group of prepubertal patients born as SGA, we also observed a negative relationship between HDL cholesterol levels and both IRI-HOMA and IRI-Belfiore. However, triglyceride levels were statistically significantly higher only in patients with hepatic IR diagnosed based on IRI-HOMA [35]. The lack of a relationship between IRI-Belfiore and triglyceride levels likely stems from the fact that the majority of patients in this group had not yet developed obesity. Lewandowski et al. [31] highlight that IRI-HOMA correlates well with the McAuley index, which is based on triglyceride levels. According to their opinion, fasting triglyceride levels can be used to assess IR in women with PCOS as an alternative to fasting glucose measurement [31].

Crucially, children who had developed peripheral IR (elevated IRI-Belfiore) but not hepatic IR (with normal IRI-HOMA) exhibited significantly higher triglyceride levels and lower HDL cholesterol levels in their serum. This may confirm that IR indices calculated based on OGTT enable the identification of a group of patients with risk factors for the development of complications related to obesity and IR before they exhibit features of metabolic syndrome (MS). It is worth mentioning that since the first definition of the MS, which was formulated in 1999 by the World Health Organization and included hyperglycemia and IR as the main criteria for diagnosing the disease, its definition has changed over time, and its criteria now include increased waist circumference, increased triglycerides, decreased HDL cholesterol, increased systolic or diastolic blood pressure, and increased fasting glucose levels [41,42,43]. Thus, IR was not always a recognized component of MS. Currently, most authors agree that the incidence of MS is increasing worldwide, and affects 20–30% of the population in developing countries, mainly due to the increasing incidence of obesity and IR [44,45]. This also applies to children, including those born small for gestational age. In our previous studies, in these groups, we detected a significant incidence of IR, among other components of MS, in the first decade of life [46]. Therefore, we believe that our research, showing the importance of the ability to recognize IR in children, is very important. 

In summary, our study findings suggest that, irrespective of the adopted criterion for diagnosing IR using IRI-HOMA, a significant percentage (26% to 46.9%) of the studied children with normal IRI-HOMA exhibited elevated IRI-Belfiore. This discrepancy arises from the fact that these indices evaluate distinct types of IR: IRI-HOMA corresponds to hepatic IR, while IRI-Belfiore represents peripheral IR. Peripheral IR typically precedes the onset of hepatic IR. Therefore, assessing IR solely through fasting glucose and insulin measurements may not capture all individuals at risk of developing type 2 diabetes and cardiovascular diseases. The higher triglyceride levels and reduced HDL cholesterol levels observed in children with IR identified through OGTT results emphasized the importance of this study and the necessity for implementing preventive measures in children.

## 5. Conclusions

1. IRI-HOMA and IRI-Belfiore values positively correlated with age, BMI, and pubertal stage, but not with the child’s gender.
2. IR was more frequently diagnosed based on the abnormal value of IRI-Belfiore than based on IRI-HOMA.
3. While the majority of children with abnormal IRI-HOMA also had an abnormal result regarding IRI-Belfiore, nearly half of the children with normal fasting insulin levels and normal IRI-HOMA exhibited increased insulin secretion during OGTT, resulting in elevated IRI-Belfiore scores.
4. In this study, children diagnosed with IR through OGTT exhibited markedly poorer lipid profiles compared to those without IR (with normal values in both IRI-Belfiore and IRI-HOMA). This finding strongly suggests that IR indices derived from OGTT facilitate the identification of individuals harboring risk factors for obesity and IR-related complications before the onset of metabolic syndrome (MS) symptoms.
5. IRI-Belfiore allows for the detection of patients with peripheral IR and increased risk factors for cardiovascular diseases even before IRI-HOMA detects liver IR.

Therefore, we present the following recommendations regarding the discussed issue:

(1) The authors of this study seek to underscore the often overlooked potential of the OGTT and the derived IRI-Belfiore in diagnosing IR. IRI-Belfiore holds promise for diagnosing IR in children at heightened risk of future cardiovascular ailments (such as those with familial predispositions to obesity, type 2 diabetes, arterial hypertension, or those born small for gestational age), especially when the widely employed IRI-HOMA yields normal results. This approach enables the early identification of peripheral IR before the onset of metabolic syndrome, facilitating the timely implementation of targeted preventive interventions.
(2) In instances where IRI-HOMA levels are elevated, assessing peripheral IR via the IRI-Belfiore index may seem superfluous, given the prevailing association of hepatic IR with peripheral IR in the majority of cases. Nevertheless, patients falling into this category should undergo OGTT to preemptively rule out disturbances in glucose tolerance or the manifestation of type 2 diabetes.

## Figures and Tables

**Table 1:** Comparison of IRI-HOMA results depending on the puberty stage and the presence of obesity in the analyzed group of children hospitalized at the Department of Endocrinology and Metabolic Diseases of the PMMH-RI between 2002 and 2018 for various reasons.

IRI-HOMA	Puberty I, *n* = 209	Puberty II and III, *n* = 111	Puberty IV and V, *n* = 233	*p*=
Normal weight, *n* = 239	0.82 ± 0.67 *, # *n* = 138	1.90 ± 1.68 * n = 31	2.34 ± 1.21 # *n* = 70	*p* = 0.00002 *, #
Obesity, *n* = 314	2.72 ± 2.11 * n = 71	3.66 ± 2.89 *n* = 80	3.79 ± 2.52 * n = 163	*p* = 0.008 *
*p*=	*p* = 0.000009	*p* = 0.002	*p* = 0.00001	

*, #—values marked with the same symbols differ significantly from each other with the given *p* value.

**Table 2:** Comparison of IRI-Belfiore results depending on the stage of pubertal maturation and the presence of obesity in the analyzed group of children hospitalized at the Department of Endocrinology and Metabolic Diseases of the PMMH-RI PMMH-RI between 2002 and 2018 for various reasons.

IRI-Belfiore	Puberty I, *n* = 209	Puberty II and III, *n* = 111	Puberty IV and V, *n* = 233	*p*=
Normal weight, *n* = 239	0.96 ± 0.36 *, # *n* = 138	1.29 ± 0.36 * n = 31	1.41 ± 0.31 # *n* = 70	*p* = 0.00002 *, #
Obesity, *n* = 314	1.38 ± 0.35 *, # *n* = 71	1.57 ± 0.28 * n = 80	1.62 ± 0.24 # *n* = 163	*p* = 0.008 *, #
*p*=	*p* = 0.000009	*p* = 0.0001	*p* = 0.000009	

*, #—values marked with the same symbols differ significantly from each other in terms of the given *p* value.

**Table 3:** Correlation between the IRI-HOMA and IRI-Belfiore and calendar age, as well as individual auxological parameters and laboratory test results, in the entire analyzed group of children hospitalized at the Department of Endocrinology and Metabolic Diseases of the PMMH-RI between 2002 and 2018 for various reasons.

	IRI-HOMA	*p*=	IRI-Belfiore	*p*=
Age (years)	0.35	0.000	0.342	0.000
HSDS	0.11	0.128	0.221	0.000
BMISDS for HA	0.537	0.000	0.532	0.000
ALT	0.307	0.000	0.197	0.000
AST	0.169	0.005	−0.153	0.799
Triglicerides	0.387	0.000	0.310	0.000
Total cholesterol	−0.009	0.837	0.453	0.323
LDL cholesterol	0.071	0.134	0.143	0.002
HDL cholesterol	−0.286	0.000	−0.273	0.000
HDL/total cholesterol	−0.284	0.000	−0.329	0.000
Glucose 0′	0.345	0.000	0.608	0.153
Glucose 60′	0.17	0.000	0.408	0.000
Glucose 120′	0.21	0.000	0.406	0.000
Insulin 0′	0.98	0.000	0.581	0.000
Insulin 60′	0.589	0.000	0.733	0.000
Insulin 120′	0.38	0.000	0.627	0.000
IRI-HOMA	-	-	0.542	0.000
IRI-Belfiore	0.542	0.000	-	-

**Table 4:** Comparison of results in children hospitalized at the Department of Endocrinology and Metabolic Diseases of the PMMH-RI between 2002 and 2018 for various reasons with a normal level of IRI-HOMA (criteria A and B) depending on whether IRI-Belfiore is at normal or elevated levels.

Normal IRI-HOMA According to Criterion A	IRI-Belfiore		
	Normal, *n* = 209 (63.1%)	Increased, *n* = 122 (36.9%)	*p*
Age (years)	10.12 ± 4.24	12.31 ± 4.35	0.00001
BMI SDS for HA	0.98 ± 2.63	2.92 ± 3.32	0.000000
Triglicerides (mg/dL)	72.84 ± 32.53	85.26 ± 37.81	0.0004
Total cholesterol (mg/dL)	160.30 ± 29.57	63.01 ± 33.43	0.480
LDL cholesterol (mg/dL)	92.67 ± 25.62	97.53 ± 30.35	0.175
HDL cholesterol (mg/dL)	53.87 ± 15.19	51.44 ± 17.14	0.239
HDL/cholesterol ratio	0.35 ± 0.10	0.32 ± 0.09	0.005
ALT (U/L)	20.31 ± 11.15	20.95 ± 8.63	0.6864
AST (U/L)	25.67 ± 8.63	23.35 ± 7.03	0.0792
**Normal IRI-HOMA** **according to criterion B**	**IRI-Belfiore**		
	**normal,** ***n* = 231 (53.1%)**	**increased,** ***n* = 204 (46.9%)**	***p***
Age (years)	10.51 ± 4.36	13.25 ± 3.88	0.000000
BMI SDS for HA	1.25 ± 2.79	3.63 ± 3.28	0.000000
Triglicerides (mg/dL)	74.98 ± 34.93	90.46 ± 41.93	0.002
Total cholesterol (mg/dL)	158.90 ± 30.78	160.45 ± 32.78	0.637
LDL cholesterol (mg/dL)	91.73 ± 26.22	97.62 ± 29.54	0.051
HDL cholesterol (mg/dL)	53.14 ± 15.08	48.22 ± 14.89	0.003
HDL/total cholesterol ratio	0.35 ± 0.10	0.31 ± 0.09	0.00005
ALT (U/L)	20.69 ± 11.59	24.71 ± 19.13	0.027
AST (U/L)	25.02 ± 8.62	23.48 ± 7.16	0.1604

**Table 5:** Comparison of study results in children hospitalized at the Department of Endocrinology and Metabolic Diseases of the PMMH-RI between 2002 and 2018 for various reasons with an elevated level of IRI-HOMA (criteria A and B) depending on whether IRI-Belfiore is at normal or elevated levels.

Increased IRI-HOMA According to Criterion A	IRI-Belfiore		
	Normal, *n* = 28 (12.6%)	Increased, *n* = 194 (87.4%)	*p*
Age (years)	14.47 ± 3.75	13.59 ± 3.23	0.184
BMI SDS for HA	3.57 ± 2.88	5.56 ± 3.20	0.002
Triglicerides (mg/dL)	92.5 ± 43.87	113.01 ± 61.42	0.103
Total cholesterol (mg/dL)	150.00 ± 35.40	160.82 ± 32.89	0.117
LDL cholesterol (mg/dL)	85.56 ± 28.40	99.42 ± 29.02	0.027
HDL cholesterol (mg/dL)	47.35 ± 12.50	44.22 ± 13.09	0.282
HDL/total cholesterol ratio	0.33 ± 0.09	0.28 ± 0.08	0.029
ALT (U/L)	21.88 ± 12.63	30.72 ± 25.78	0.0762
AST (U/L)	22.17 ± 7.87	26.95 ± 14.41	0.2208
**Increased IRI-HOMA according to criterion B**	**IRI-Belfiore**		
	**normal,** ***n* = 6 (5.1%)**	**increased,** ***n* = 112 (94.9%)**	***p***
Age (years)	15.42 ± 4.11	12.80 ± 3.48	0.077
BMI SDS for HA	2.84 ± 2.02	6.20 ± 3.26	0.014
Triglicerides (mg/dL)	89.83 ± 26.91	123.55 ± 68.33	0.233
Cholesterol (mg/dL)	159.67 ± 22.62	163.77 ± 33.59	0.769
LDL cholesterol (mg/dL)	90.80 ± 21.11	100.59 ± 29.41	0.465
HDL cholesterol (mg/dL)	49.50 ± 12.37	44.54 ± 15.22	0.523
HDL/total cholesterol ratio	0.30 ± 0.06	0.28 ± 0.08	0.545
ALT (U/L)	18.6 ± 2.5	31.26 ± 7.2	0.2685
AST (U/L)	23.0 ± 0.0	28.25 ± 17.59	0.6762

## Data Availability

Data are contained within the article.
